# A randomized controlled trial of the effect of supervised progressive cross-continuum strength training and protein supplementation in older medical patients: the STAND-Cph trial

**DOI:** 10.1186/s13063-019-3720-x

**Published:** 2019-11-28

**Authors:** Mette Merete Pedersen, Janne Petersen, Nina Beyer, Helle Gybel-Juul Larsen, Pia Søe Jensen, Ove Andersen, Thomas Bandholm, on behalf of the STAND-Cph collaborative group, Ove Andersen, Thomas Bandholm, Jonathan F. Bean, Nina Beyer, Ann Christine Bodilsen, Rasmus Brødsgaard, Jette Christensen, Line Due Christensen, Christina Dahl, Lars Damkjær, Simon Hegnsvad, Pia Søe Jensen, Karen Dalsgaard Jepsen, Helle Gybel Juul-Larsen, Louise Lawson-Smith, Morten Ledertoug, Robert-Jan Nienhuis, Sanne Stiberg, Maja Pedersen, Mette Merete Pedersen, Frida Margareta Persson, Janne Petersen, Trine Rehfeld Lind, Rikke Friis Knudsen, Gitte Salomonsen, Bettina Solgaard, Lisbeth Sommer, Maria Therese Stage, Berit Thorslund, Marie West, Jade Kavanaugh Wulff

**Affiliations:** 1 Clinical Research Centre, Copenhagen University Hospital Hvidovre, Kettegaard Alle 30, 2650 Hvidovre, Denmark; 2 Section of Biostatistics, Department of Public Health, University of Copenhagen, Øster Farimagsgade 5 Entrance B, 2nd floor, 1014 Copenhagen K, Denmark; 3 Center for Clinical Research and Prevention, Copenhagen University Hospital, Bispebjerg and Frederiksberg, Bispebjerg Bakke 23, 2400 Copenhagen N, Denmark; 4 Department of Physical and Occupational Therapy, Copenhagen University Hospital, Bispebjerg and Frederiksberg, Bispebjerg Bakke 23, 2400 Copenhagen N, Denmark; 5 Department of Clinical Medicine, Faculty of Health and Medical Sciences, University of Copenhagen, Blegdamsvej 3B, 2200 Copenhagen N, Denmark; 6 Department of Orthopaedic Surgery, Copenhagen University Hospital, Kettegaard Alle 30, 2650 Hvidovre, Denmark; 7 The Emergency Department, Copenhagen University Hospital, Kettegaard Alle 30, 2650 Hvidovre, Denmark; 8 Department of Physical- and Occupational Therapy, Copenhagen University Hospital Hvidovre, Kettegaard Alle 30, 2650 Hvidovre, Denmark

**Keywords:** Older medical patients, Strength training, Cross-continuum, Mobility, Activity

## Abstract

**Background:**

During hospitalization, older adults (+ 65 years) are inactive, which puts them at risk of functional decline and loss of independence. Systematic strength training can prevent loss of functional performance and combining strength training with protein supplementation may enhance the response in muscle mass and strength. However, we lack knowledge about the effect of strength training commenced during hospitalization and continued after discharge in older medical patients. This assessor-blinded, randomized study investigated the effect of a simple, supervised strength training program for the lower extremities, combined with post-training protein supplementation during hospitalization and in the home setting for 4 weeks after discharge, on the effect on change in mobility in older medical patients.

**Methods:**

Older medical patients (≥ 65 years) admitted acutely from their home to the Emergency Department were randomized to either standard care or supervised progressive strength training and an oral protein supplement during hospitalization and at home 3 days/week for 4 weeks after discharge. The primary outcome was between-group difference in change in mobility from baseline to 4 weeks after discharge assessed by the De Morton Mobility Index, which assesses bed mobility, chair mobility, static and dynamic balance, and walking. Secondary outcomes were 24-h mobility, lower extremity strength, gait speed, grip strength and activities of daily living.

**Results:**

Eighty-five patients were randomized to an intervention group (*N* = 43) or a control group (*N* = 42). In the intervention group, 43% were highly compliant with the intervention. Our intention-to-treat analysis revealed no between-group difference in mobility (mean difference in change from baseline to 4 weeks, − 4.17 (95% CI − 11.09; 2.74; *p* = 0.24) nor in any of the secondary outcomes. The per-protocol analysis showed that the daily number of steps taken increased significantly more in the intervention group compared to the control group (mean difference in change from baseline to 4 weeks, 1033.4 steps (95% CI 4.1; 2062.7), *p* = 0.049, adjusted for mobility at baseline and length of stay; 1032.8 steps (95% CI 3.6; 2061.9), *p* = 0.049, adjusted for mobility at baseline, length of stay, and steps at baseline).

**Conclusions:**

Simple supervised strength training for the lower extremities, combined with protein supplementation initiated during hospitalization and continued at home for 4 weeks after discharge was not superior to usual care in the effect on change in mobility at 4 weeks in older medical patients. For the secondary outcome, daily number of steps, high compliance with the intervention resulted in a greater daily number of steps. Less than half of the patients were compliant with the intervention indicating that a simpler intervention might be needed.

**Trial registration:**

ClinicalTrials.gov, NCT01964482. Registered on 14 October 2013. Trial protocol PubMed ID (PMID), 27039381.

## Background

For many older adults, the ability to live independently is ranked a very important health outcome [1], and independence is considered a prerequisite for control and freedom of choice in daily life [2]. Therefore, it is unfortunate that for many older adults (+ 65 years) hospitalization is linked with an increased risk of mobility limitations, functional decline, and loss of independence [3–5], partly due to preventable events like excessive bed rest or low levels of mobility [6–10]. Nevertheless, older adults spend the majority of their in-hospital time in bed [8, 11–15].

The adverse events due to in-hospital inactivity include loss of muscle mass and strength [16–21], especially in the lower extremities [17, 20, 22, 23]. This loss is very rapid and compared to young adults it takes longer for older adults to regain the loss [24], and impairments in muscle strength and power are influential determinants of mobility problems and disability in older adults [25, 26].

It has been reported that functional status at discharge and one month after discharge are associated with long-term outcomes [27, 28]. Thus, it seems imperative to reduce physical inactivity during hospitalization and to focus on regaining functional performance within the first month after discharge, preferably by initiating an exercise program in the hospital and continuing this program in the home setting after discharge [29–31].

Systematic strength training has shown promising results in preventing loss of strength and functional performance [32–36] and in improving mobility in frail older adults [34] Also, weight-bearing exercises seem preferable to non-weight-bearing exercises [37] and higher intensities superior to lower intensities [38–41]. However, there is a lack of knowledge on the optimal dose and intensity of strength training and the optimal exercises in different settings for older adults [32, 38, 42], and challenges with compliance have been reported [29, 43, 44]. Moreover, combining strength training with protein supplementation may stimulate muscle protein synthesis and thus increase the effects of the response to exercise on muscle mass and strength as seen in healthy older adults [45–47].

Therefore, in this randomized controlled trial in older medical patients, we determined the effect on mobility of a simple, low-technology, supervised, high-intensity, strength training program for the lower extremities, combined with post-training protein supplementation initiated during hospitalization and continued in the home setting for 4 weeks after discharge. We hypothesized that the intervention was superior to usual care.

## Methods

### Study design and participants

This is the primary trial report of a randomized controlled trial conducted from September 2013 to September 2016 at Copenhagen University Hospital Hvidovre in Denmark and in the participants’ own homes (ClinicalTrials.gov identifier, NCT01964482). Patients from three municipalities (Copenhagen, Hvidovre and Broendby) were recruited on week days except during holiday periods. During this period, recruitment was paused for 7 months in the municipality of Copenhagen (January to July 2014) and for 1 month in the municipality of Hvidovre (June 2015) due to lack of staff. A full trial protocol - published before inclusion of the last participant - is available with open access [48]. Briefly, on week days the primary investigator or one of three assistant investigators identified eligible newly admitted patients. Older medical patients (≥ 65 years) admitted with acute illness from their own home to the Emergency Department of the hospital were included based on random sampling. Patients were excluded on the following criteria: terminal illness; in treatment for diagnosed cancer; diagnosis of Chronic Obstructive Pulmonary Disease (COPD) and participation in a COPD rehabilitation program; living outside the three included municipalities; inability to speak or understand Danish; inability to cooperate in tests/exercises; transfer to the intensive care unit; isolation-room stay; expected hospitalization lasting < 24 h; or inability to stand. The reporting of this study follows the Consolidated Standards of Reporting Trials (CONSORT) statement, using the extension for non-pharmacological trials [49] and the Consensus on Exercise Reporting Template (CERT) checklist [50].

### Procedures

#### Assessments and randomization

After inclusion, baseline assessments were performed by one of four outcome assessors at the Acute Medical Admissions Ward or at an internal medicine ward at the hospital. Hereafter, the patients included were randomized to either the intervention group or the control group according to a computer-generated block randomization list developed by the study coordinator [46]. The patients were reassessed in their own homes within the first week following discharge, 4–5 weeks after discharge (primary endpoint and end of intervention) and 6 months after discharge. The primary investigator or one of the three assistant investigators, who are all trained physiotherapists, were outcome assessors and performed all baseline and follow-up assessments. All assessments of a patient were performed by the same investigator whenever logistically possible.

#### Blinding

We ensured that the outcome assessors were blinded to group assignment [46] and the randomization list was unavailable to the outcome assessors at all times. The research assistant handled all communication with physiotherapists in charge of supervising the strength training sessions and all logistics in connection with home assessments. Also, all patients were asked not to reveal to the investigators to which group they belonged.

### Study groups

#### Control group

Patients assigned to the control group received standard care during hospitalization and following discharge (for further details, please see Pedersen et al. 2016 [48]).

#### Intervention group

**Fig. 1:**
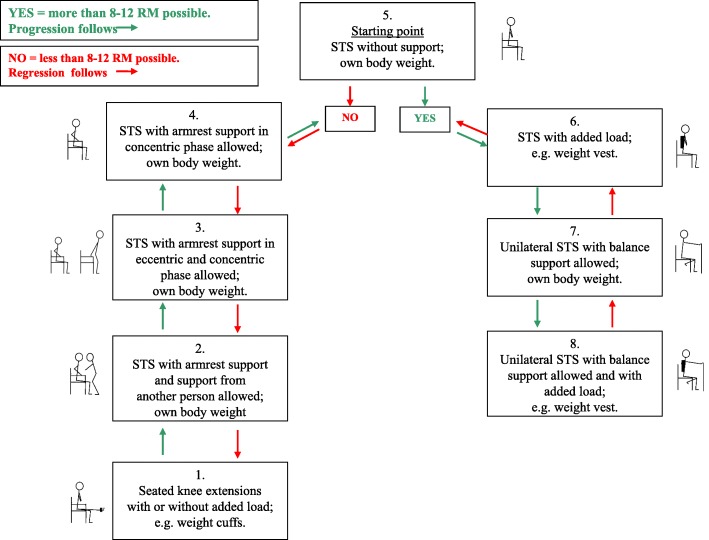
Progression model for loaded sit-to-stand exercise (STAND). The starting point in STAND in the first session was level 5. The patient was seated in a standard chair with armrests with the feet on the floor at shoulder-width apart and arms crossed at the wrist with the hands placed on the opposite shoulder. The patient was asked to rise to a fully extended position and to sit down at a constant pace and was encouraged verbally to perform as many repetitions as possible. The supervising physiotherapist ensured that each set of the exercise was performed at a level of the model ensuring 8–12 repetition maximum (RM). If extra weight was needed, a weight vest (Titan Box, 1–30 kg) was used. STS, 30-s sit-to-stand test. The stick art is the author’s own work and was published for the first time in Pedersen et al. PeerJ (2015) 3:e1500; DOI 10.7717/peerj.1500in and subsequently in Pedersen et al. Trials (2016) 17:176)

**Fig. 2:**
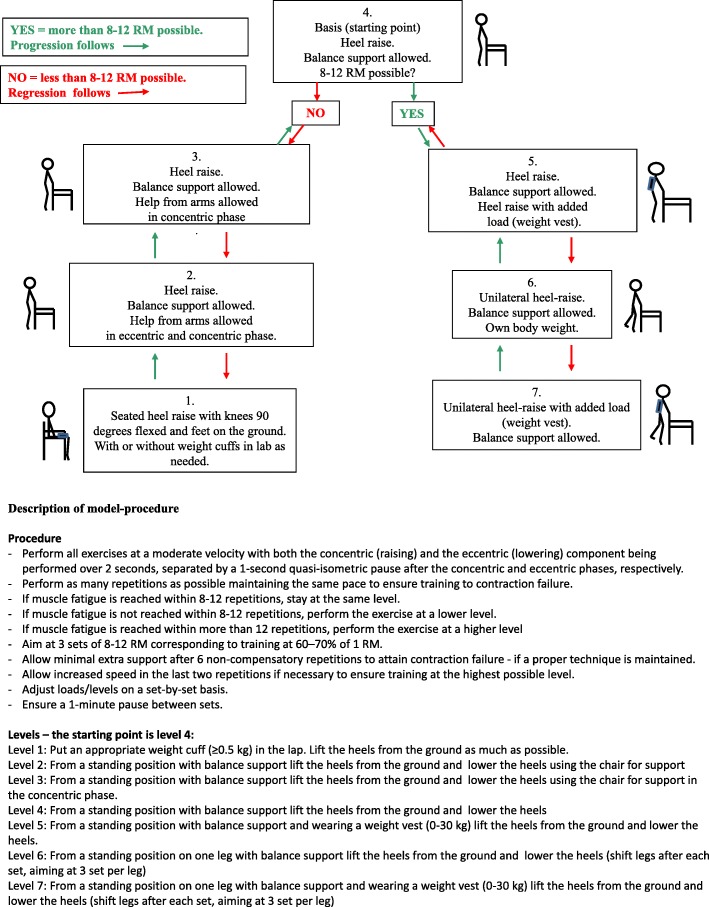
Progression model for loaded heel-raise (heel-raise). The starting point in heel-raise in the first session was level 4. The patient was standing behind a standard chair using the chair for balance support and keeping the feet on the floor at shoulder-width apart. The patient was asked to lift both heels to stand on the forefoot and to lower the heels to a standing position at a constant pace, and was encouraged verbally to perform as many repetitions as possible. The supervising physiotherapist ensured that each set of the exercise was performed at a level of the model ensuring 8–12 repetition maximum (RM). If extra weight was needed, a weight vest (Titan Box, 1–30 kg) was used. The stick art is the author’s own work and was published for the first time in Pedersen et al. Trials (2016) 17:176)

Patients assigned to the intervention group received progressive strength training supervised 1:1 by a skilled physiotherapist on week days during hospitalization and 3 days per week for 4 weeks in their own home after discharge, since recovery of functioning within the first month after discharge has been shown to be important for long-term outcomes [27]. A total of 12 training sessions (over a maximum of 5 weeks) were provided after discharge. Every training session consisted of a warm-up program for the lower extremities followed by two progressive strength training exercises, a sit-to-stand exercise (STAND) (Fig. 1) followed by a heel raise exercise (heel-raise) (Fig. 2) as outlined in Pedersen et al. 2016 [48]. Both exercises followed predefined models of progression allowing for performance of the exercise from a seated position to performing the exercise unilaterally with extra load (level 1 to level 7/level 8). The STAND progression model was found feasible in a previous study [51]. Both exercises were performed for three sets of 8–12 repetitions to fatigue in each set (8–12 repetition maximum (RM)) [52]. The progression models shown in Figs. 1 and 2 were applied to every single set of both exercises by the supervising physiotherapist. Each training session lasted approximately 20 min including warm up. Immediately after each training session the patients were asked to consume an oral protein supplement (Nutridrink Compact Protein from Nutricia A/S) containing 18 g milk-based protein and 300 kcal.

##### Standardization of assessments and intervention

To ensure standardization of the intervention, the primary investigator performed pre-intervention meetings with all involved outcome assessors and physiotherapists. At meetings for outcome assessors only, the assessors were introduced to and trained in the assessments to ensure standardization. In addition, before conducting an assessment the outcome assessors observed one or two sessions conducted by the primary investigator. Further, the primary investigator supervised each assessor for an assessment at the hospital and an assessment in a home setting. At meetings with physiotherapists only, the physiotherapists were trained in both the warm-up program and the strength training protocol. Should questions arise during the study, the outcome assessors could contact the primary investigator about the assessments and the physiotherapists could contact a senior physiotherapist about the exercise intervention. Also, a geriatrician could be contacted in the case of medical concerns.

### Outcomes

The outcome assessments were performed on admission (baseline), within the first week after discharge, 4–5 weeks after discharge (primary endpoint), and 6 months after discharge.

#### Primary outcome measure

The primary outcome was change in the De Morton Mobility Index (DEMMI) score from baseline to 4 weeks after discharge. The DEMMI is a valid and reliable measure of mobility in older adults assessing bed mobility, chair mobility, static and dynamic balance, and walking [53–56]. The DEMMI is scored from 0 to 100 where 100 represents the highest level of mobility [53, 54] and a score below 62 is considered to reflect limited mobility in community-dwelling older adults [57]. In acute older medical patients, the minimal clinically important difference on the DEMMI score is 10 points [53, 56].

#### Secondary outcome measures

The secondary outcomes have been described in detail in Pedersen et al. 2016 [48]). Briefly, the secondary outcomes were:

1. The 24-h mobility measured by an *activ*PAL3™ activity monitor (PAL Technologies Ltd., Glasgow, UK). The patient wore the *activ*PAL3™ from inclusion into the study and throughout the entire hospitalization, the first week after discharge, the first week after the 4-week assessment and the first week after the 6-month assessment. The *activ*PAL3™ monitors continuously for 7 days and assesses time spent sitting/lying, standing, and walking, and the number of steps taken. During hospitalization, the monitor was replaced after 7 days in the case of hospitalizations extending beyond 7 days. In agreement with an observational study preceding this randomized controlled study [11], and to maximize the number of full days with 24 h of measurements, we considered a day to be from 12:00 a.m. until 12:00 a.m. to avoid half-day measurements as the accelerometers were normally attached in the morning. Because very few patients are hospitalized for more than 6 days, we only included the first 6 days of hospitalization in the analysis. To avoid skewed days in the analysis, we included only patient-days with more than 20 h of measurements when studying the distributions of sitting/lying, standing, and walking. The ActivPal3™ has been shown valid and reliable in measuring posture and transitions in mobility-limited older adults [58, 59] and in measuring walking at speeds between 0.67 m/s and 1.56 m/s [60–62]. The ActivPal3™ data will be dichotomized into sedentary (sitting/lying) and upright time (walking/standing) according to our protocol [48] if 15% walk at speeds below 0.67 m/s, since the percentage error in measuring steps is greater for slow walkers than fast walkers [59–63].
2. Isometric knee extension strength (IKE; Nm/kg) in the dominant leg measured by a handheld dynamometer (Power Track II Commander; JTech Medical, Utah) with the patient seated in a standard chair (height 45 cm), arms crossed over the chest and 90° knee flexion [64, 65]. The patient was asked to perform three maximal knee extensions (5 s duration, 1 min apart). The highest value obtained was used as the outcome.
3. The 30-s sit-to-stand test (STS; number performed in 30 s) using a standard arm chair (seat height 45 cm) [66]. The patient was asked to stand up and sit down as many times as possible in a 30-s period. We used a modified 30-s STS allowing arm rest support for patients who were unable to rise once from the chair with the arms crossed over the chest.
4. Habitual gait speed (HG; m/s) measured on a 4-m course [67, 68] from a standing start position (walking aids were allowed). The faster of two walks was used as the outcome. A gait speed below 0.8 m/s is considered to reflect poor mobility [69].
5. Hand-grip strength (HGS; kg) in the dominant hand measured with a handheld dynamometer (Digi-II; Saehan) with the patient seated in a standard armchair (seat height 45 cm), with the lower dominant arm placed on the armrest, 90° elbow flexion and neutral wrist position. The highest value of three maximal squeezes of the handle (5 s duration, 1 min apart) was used as the outcome.
6. The Barthel Index 20 (BI) was used as a measure of activities of daily living (ADL) [70]. The BI is scored between 0 and 20 with higher scores indicating less disability in ADL.

#### Additional variables

We collected descriptive variables and possible confounders and modifiers: age, sex, education, living status, history of smoking, use of ambulatory devices, use of municipal help, history of falls during the last year, mobility assessed by the New Mobility Score (NMS) [71, 72], ambulatory capacity assessed by the Cumulated Ambulation Score [73], cognition assessed by the Short Orientation-Memory-Concentration test (OMC) [74] and the Mini Mental State Examination [75], depression assessed by the Geriatric Depression Scale (GDS) [76], health status assessed by the EuroQol instrument [77], nutritional state assessed by the Mini Nutritional Assessment [78], self-reported physical activity [79, 80], pain before and after training assessed by the Verbal Ranking Scale (VRS) [81, 82], medications, history of training before admission, and history of municipal training after discharge. In addition to the protocol [48], from January 2015 patients in the intervention group were asked about their satisfaction with the strength training intervention in 5 questions with corresponding 3–4-level rating scales: (1) How satisfied are you with the training intervention? (“Very satisfied”; “Satisfied”; “Dissatisfied”; “Very dissatisfied”; “Don’t know”); (2) I have benefitted from the strength training sessions (“Strongly agree”; “Agree; “Disagree; “Strongly disagree; “Don’t know); (3) The amount of training was (“Appropriate”; “Too little”; “Too much”; “Don’t know”); (4) I will (“Continue training on my own”; “Continue training with others”; “Stop training”; “I don’t know”); (5) Are you satisfied with the results of the training? (“Very satisfied”; “Satisfied”; “Dissatisfied”; “Very dissatisfied”; “I don’t know”). A research assistant called the participant after completion of the intervention to ask these questions. Also, from the National Patient Registry we obtained data on comorbidities and readmissions. The Charlson Comorbidity Index [83] was calculated based on the *International Classification of Diseases, 10th Edition* (ICD-10) [84], and we used registry data on hospital admissions and outpatient visits during the 10 years preceding the admission that was related to inclusion in this study.

##### Exercise diary

During each training session the supervising physiotherapist recorded the exercise level, number of repetitions completed, and the extra load added (in kilograms). Pain before and after each training session was recorded using the VRS [81]. The amount of protein consumed and reasons for non-participation were also recorded. Good compliance with the intervention was defined as completion of 80% of all training sessions with a minimum of two sets performed per session and moderate compliance was defined as completion of 67% of all training sessions (8 out of 12 sessions) performed with a minimum of two sets per session.

### Data management

Trial data management complied with the rules of the Danish Data Protection Agency and was performed blinded to group allocation. All data were double-entered in Epidata Entry 3.1 (Epidata Associations, Odense, Denmark). Ranges were checked for data values and checked against the case report forms. Data from the *activ*PAL3™ monitors were downloaded using the *activ*PAL™ Professional software version 7.2.32. All data were exported to SAS Enterprise Guide 7.1 (SAS Institute Inc., Gary, NC, USA).

### Sample size

We estimated our sample size based on unpublished data from a cohort study performed at Hvidovre Hospital in older medical patients, showing mean change in the de Morton Mobility Index [56] score of 1.8 from admission to 4 weeks after discharge and standard deviation of 12.8. A sample of 54 patients was required to detect a 10-point difference (minimal clinically important difference [56]) in the between-group change in the DEMMI score at the 4-week assessment, given a type I error rate of 5% and power of 80% for a two-sample *t* test of a normal mean difference with a two-sided significance level. According to our protocol, we included patients until both groups contained 25 patients assessed for the primary endpoint (4 weeks).

### Statistical analyses

Depending on the distribution of the variables, descriptive data are presented either as means with standard deviations, medians with interquartile ranges or frequencies with percentages. For determining differences between participants who remained in the study and participants who dropped out we used the chi squared (χ^2^) test to test for difference in sex and Student’s *t* test to test for differences in age and the DEMMI score. Our primary analysis for the primary outcome was a mixed model analysis of the between-group difference in change in the DEMMI score from baseline to 4 weeks after discharge using the SAS procedure PROC MIXED. All randomized patients were analyzed following the intention-to-treat (ITT) principle, using multiple imputation in the case of missing data points, and the analysis was unadjusted (for the number of imputations at each assessment point, please see Additional file 1). We used a fully conditional specification (FCS) regression method in PROC MI for imputation of missing data based on age, group, and all previous assessments or time points. For ActivPal data, only the first day during hospitalization was imputed to avoid imputation for days when the patients were not hospitalized and thereby to account for differences in lengths of stay. This imputation was based on age, group, and baseline DEMMI score. According to the method of Graham et al. [85], we used 100 imputations to avoid a power falloff. The patient identification number and municipalities were modeled as random variables, group and time were modelled as fixed factors, and the between-group difference in change in the DEMMI score was estimated from the interaction between the time and the group variable. We also analyzed the effect during hospitalization and the effect after the intervention ended, based on the primary analysis model. Similar analyses were performed for all outcomes with all analyses adjusted for baseline DEMMI score to adjust for the large variation in mobility in older medical patients [86]. Also, to account for imbalances in time spent in hospital, the effect during hospitalization and the effect from baseline to the end of the intervention adjusted for length of hospital stay were analyzed. In addition, a per-protocol (PP) analysis was performed comparing patients who had fulfilled the compliance criteria (80% of post-discharge sessions completed with a minimum of two sets in each session) with those in the control group who had not dropped out before the 4-week assessment and, thus, would have been able to comply with the intervention if they had been assigned to the intervention group. In the PP analysis, ActivPal data from days 5 and 6 at 6 months were not included in the analysis due to too many missing data. In supplement to the aforementioned analyses, all ITT and PP analyses were conducted with adjustment for baseline values of the outcome of interest. All between-group differences are expressed as the average difference in change from baseline to relevant outcome time with corresponding 95% confidence intervals. All models were investigated for goodness-of-fit (linearity, variance homogeneity, and normal distribution of residuals) by visual inspection of residual plots and were remodeled if necessary. We used SAS Enterprise Guide 7.1 (SAS Institute Inc., Gary, NC, USA) for all statistical analyses and considered *p* values ≤0.05 to be statistically significant. However, for all analyses evaluating potential modifiers and confounders of the intervention, *p* values ≤0.01 were used to account for multiple testing.

## Results

**Fig. 3:**
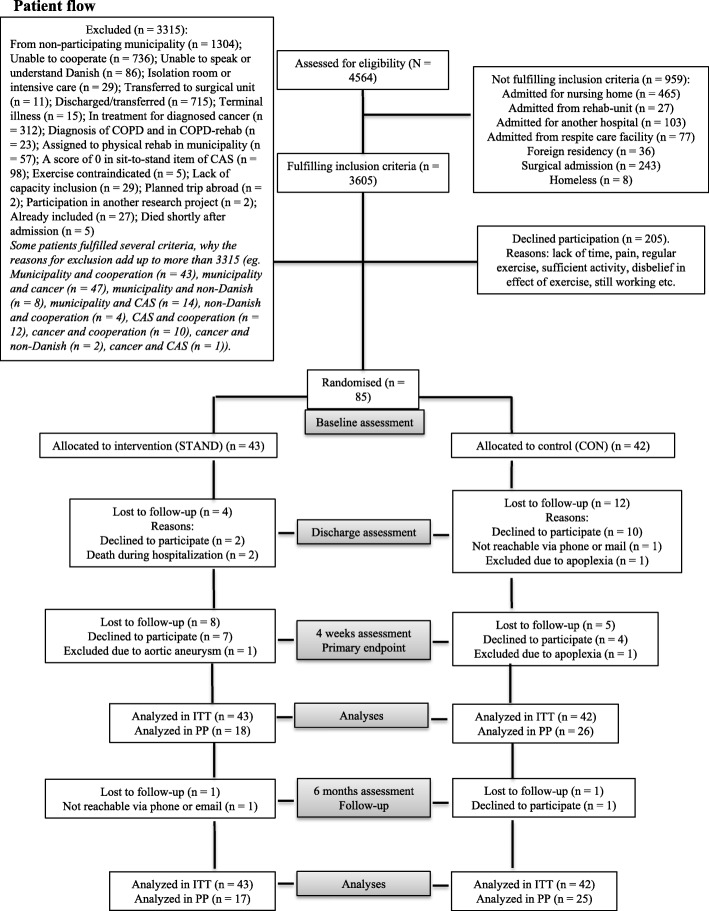
Flow of patients. COPD, chronic obstructive pulmonary disease; CAS, Cumulated Ambulation Score; STAND, sit-to-stand exercise; ITT, intention to treat; PP, per protocol

A total of 85 patients were included and randomized to the intervention group (*N* = 43) or the control group (*N* = 42). Figure 3 illustrates the flow of patients throughout the study [49]. Between baseline and 4 weeks, twelve patients in the intervention group were lost to follow up (27%). Reasons for declining to participate any further were lack of information about the extent of the study; lack of time/too many things going on; being in the middle of a divorce; exercise-induced muscle soreness; and chronic leg pain. Seventeen patients in the control group were lost to follow up (40%). Reasons for declining to participate any further were impeding surgery; not wishing to continue; lack of time/too many things going on; lack of information; and not wanting to have anything to do with the hospital.

There were no significant baseline differences in age, sex, or DEMMI score between patients lost to follow up and patients remaining in the study at 4 weeks (all *p* > 0.31). One patient in each of the two groups was lost to follow up between 4 weeks and 6 months. The ActivPal3™ data was missing for 24 patients during hospitalization (8 patients were lost to follow up, in 2 patients the monitors were not attached due to miscommunication, 1 patient had an allergic reaction to the adhesive padding, 7 patients had monitors lost on the ward, and 6 patients had data for less than 20 h); for 29 patients after discharge (2 patients took the monitor off, 1 patient had an allergic reaction to the adhesive band, 14 patients were lost to follow up, 10 patients did not want to wear the monitor, 1 patient experienced itching under the monitor, and 1 patient lost the monitor); for 41 patients after the 4 week assessment (27 patients were lost to follow up, 2 patients were not able to cooperate, 1 patient was allergic to the adhesive band, 10 patients did not want to wear the monitor, and 1 patient experienced itching under the monitor); and 46 patients after the 6 months assessment (29 patients were lost to follow up, 2 patients were not able to cooperate, 1 patient was allergic to the adhesive band, 12 patients did not want to wear the monitor, 1 patient experienced itching under the monitor, and 2 patients lost the monitors).

### Baseline characteristics

At baseline, no between-group differences appeared to be clinically relevant (no hypothesis testing was undertaken as suggested by the CONSORT group, Table 1). Overall, the patients were 82.3 years of age (SD 7.4), 65.9% were female, 67.1% were living alone, and 54% had an NMS reflecting poor function independency (a score of 0–5) [71]. The majority was admitted to the hospital with pulmonary problems and the median length of stay (LOS) was 4 days.

**Table 1:** Baseline characteristics

Descriptive data	Overall (*N* = 85)	Intervention (*N* = 42)	Control (*N* = 43)
Age (years)	82.3 (7.4)	82.1 (7.4)	82.5 (7.5)
Sex			
Male	34.1%	28.6%	39.5%
Female	65.9%	71.4%	60.5%
BMI	25.3 (22.3;29.3)	25.3 (22.3;29.1)	24.5 (22.3;30.0)
Living alone (yes, %)	67.1%	69.1%	65.1%
Education (%)			
< High school	25.9%	23.8%	27.9%
Skilled	55.3%	54.8%	55.8%
High school	3.5%	4.8%	2.3%
Graduate	9.4%	9.5%	9.3%
Post graduate	5.9%	7.1%	4.7%
Smoking status			
Smoking (yes, %)	17.7%	19.1%	16.3%
Previous smoker (yes, %)	81.2%	78.6%	83.7%
Assistive devices			
Walking stick	25.9%	14.3%	37.2%
Crutches	8.2%	9.5%	6.9%
Walker	34.1%	28.6%	39.5%
Wheel chair	3.5%	4.8%	2.3%
Furniture support	28.6%	29.3%	27.9%
Scooter	5.9%	2.4%	9.3%
Use of municipal help			
Assistance from community (yes, %)	62.4%	61.9%	62.8%
Personal help (yes, %)	11.8%	19.1%	4.7%
Cleaning (yes, %)	20.0%	16.7%	23.3%
Fall during past year (yes, %)	51.8%	52.4%	51.2%
Short Falls Efficacy Scale (score)	8 (7;10)	8 (7;11)	8 (7;9)
Admission diagnosis (category)			
Pulmonary	43.5%	45.2%	41.9%
Cardiovascular	25.9%	21.4%	30.2%
Other^a^	30.6%	33.3%	27.9%
Charlson Comorbitity Index (*n*)			
0	18.8%	21.4%	16.3%
1–2	52.9%	47.6%	58.1%
3+	28.2%	31.0%	25.6%
Length of stay (days)	4 (2;7)	4.5 (2.5;7.0)	4 (2;6)
New Mobility Score (points)			
Admission	5 (2;7)	4 (2;7)	5 (2;9)
In retrospect	7 (5;9)	7 (6;9)	6 (5;9)

Variables are presented as mean (SD), median (IQR) or percentages depending on the distribution of the variable.

*BMI* body mass index

^a^ Endocrinological, neurological, hepato-nephrological, gastrological, dermatological

### Outcomes

At baseline, the participants had an average DEMMI score of 60.7 (SD 16.4) reflecting limited mobility [57]. The between-group differences for the primary and secondary outcome measures are shown in Table 2, and the values for the primary and secondary outcomes at the four assessment points are shown in Table 3.

**Table 2:** Between-group differences in change scores from baseline to discharge, 4 weeks, and 6 months (∆intervention-∆control)

Primary outcome	Baseline to 4 weeks	*P*	Baseline to discharge	*P*	4 weeks to 6 months	*P*
DEMMI, score (ITT)	−4.17 (−11.09;2.74)	0.24	−0.51 (−6.51;5.49)	0.87	2.97 (−2.81;8.76)	0.31
DEMMI, score (PP)	−1.00 (−9.28;7.27)	0.81	4.70 (−3.30;12.70)	0.25	1.55 (−2.86;5.95)	0.49
Secondary outcomes						
24-h activity measures (ActivPal)						
Upright time, h/day (ITT)	0.06 (−0.89;1.02)	0.90	−0.59 (−1.16;0.01)	0.046	0.21 (−1.18;160)	0.77
Upright time, h/day (PP)	0.25 (−0.61;1.11)	0.57	−0.55 (−1.14;0.05)	0.07	−0.002 (−1.36;1.37)	1.00
Lying/sitting, h/day (ITT)	0.03 (−1.02;1.09)	0.95	0.46 (−0.21;1.14)	0.18	−0.001 (−1.35;1.34)	0.99
Lying/sitting, h/day (PP)	−0.09 (−1.08;0.90)	0.86	0.28 (−0.42;0.98)	0.44	−0.19 (−1.45;1.07)	0.77
Steps, *n* (ITT)	472.48 (− 536.44;1481.41)	0.36	117.76 (−465.37;700.90)	0.69	339.12 (−1152.00;1830.25)	0.65
Steps, *n* (PP)	999.19 (−23.88;2022.25)	0.05	303.02 (− 272.84;878.88)	0.30	44.59 (− 1658.60;1747.79)	0.96
Adjusted for DEMMI and LOS	1033.40 (4.13;2062.66)	0.049				
Adjusted for DEMMI, steps, and LOS	1032.77 (3.60;2061.94)	0.049				
Physical performance measures						
KES, Nm/kg (ITT)	0.11(−0.02;0.24)	0.09	0.08 (−0.05;0.20)	0.24	−0.09 (− 0.20;0.02)	0.11
KES, Nm/kg (PP)	0.13 (−0.02;0.29)	0.09	0.10 (−0.07;0.27)	0.23	−0.11 (− 0.23;-0.002)	0.047
STS, *n* (ITT)	0.04 (−1.88;1.96)	0.97	0.33 (−1.22;1.87)	0.68	1.28 (−0.76;3.31)	0.22
STS, *n* (PP)	−0.01(−2.29;2.27)	1.00	0.21(−1.96;2.39)	0.85	1.45 (−0.94;3.84)	0.23
GS, m/s (ITT)	0.02(−0.06;0.10)	0.64	0.07(−0.003;0.15)	0.06	0.02(−0.06;0.11)	0.57
GS, m/s (PP)	0.04(−0.07;0.15)	0.45	0.11(0.004;0.22)	0.04	0.02(−0.08;0.11)	0.74
HG, kg (ITT)	0.49(−1.40;2.39)	0.61	1.86 (0.49;3.23)	0.008	0.17 (−1.46;1.80)	0.84
HG, kg (PP)	0.75(−1.37;2.87)	0.48	2.05(0.32;3.77)	0.02	−0.28(−2.11;1.55)	0.76
Barthel, score (ITT)	−0.09 (− 0.83;0.66)	0.82	− 0.26 (− 0.87;0.34)	0.40	0.30 (− 0.11;0.70)	0.16
Barthel, score (PP)	0.44 (− 0.42;1.31)	0.30	0.02 (− 0.65;0.70)	0.94	0.20 (− 0.20;0.61)	0.33

Unadjusted values are shown for all comparisons and adjusted values are shown where these differ from the unadjusted analyses. All values are given as mean (95% confidence interval). For the number of imputations at different time points for the primary and secondary outcomes please see Additional file 1

*ITT* intention to treat (*N* = 85), *PP* per protocol (*N* = 18 in intervention group (only those compliant with the intervention); *N* = 26 in control group), *DEMMI* baseline value of De Morton Mobility Index, *steps*: baseline value of steps, *LOS* length of stay, *KES* knee extension strength, *STS* 30-s sit-to-stand, *GS* gait speed, *HG* hand grip strength, *Barthel* Barthel Index

**Table 3:** Primary and secondary outcomes at four assessment points (ITT) (non-imputed data)

	*N*	Hospitalization	*N*	Discharge	*N*	4 weeks	*N*	6 months
	Intervention (*N* = 42)							
De Morton Mobility Index (points)	42	63.5 (16.4)	37	69.8 (16.5)	30	71.9 (15.7)	27	70.9 (15.5)
24-h mobility (h/day)								
Lying/sitting	36	21.8 (1.2)	31	20.6 (1.3)	25	19.7 (1.8)	22	19.4 (1.9)
Uptime	36	1.4 (01.0;1.8)	31	3.2 (1.8;3.7)	25	4.0 (3.0;5.2)	22	4.1 (2.7;5.3)
Standing	36	1.2 (0.8;1.7)	31	2.5 (1.5;3.1)	25	2.8 (2.4;4.5)	22	3.1 (2.2;4.4)
Walking	36	0.2 (0.1;0.3)	31	0.5 (0.3;0.8)	25	0.9 (0.4;1.0)	22	0.9 (0.4;1.1)
Steps (*n*/day)	36	660 (264;1285)	31	1962 (1114;3219)	25	4147 (1471;4683)	22	3515 (1751;4805)
Transitions (*n* up-down/day)	36	50 (41;82)	31	57 (46;78)	25	54 (47;64)	22	56 (43;78)
Isometric knee extension (Nm/kg)	42	0.7 (0.5;0.9)	36	0.7 (0.5;0.9)	29	0.8 (0.6;1.0)	26	0.8 (0.6;0.9)
30-s Sit-to-stand test (reps)	40	5.5 (0 9.5)	37	8 (5;11)	30	9.5 (6;12)	27	10 (5;12)
30-s Sit-to-stand test mod. (reps)^a^	11	5 (2;7)	6	6.5 (5;8)	5	4 (4;9)	5	7 (4;8)
Habitual Gait Speed (m/s)	41	0.6 (0.4;0.8)	37	0.7 (0.6;0.9)	30	0.7 (0.6;0.9)	27	0.7 (0.6;0.9)
Hand grip strength (kg)	42	21.5 (10.3)	38	23.5 (9.9)	30	23.5 (10.0)	27	24.0 (10.2)
Barthel Index 20 (points)	42	20 (19;20)	37	20 (19;20)	30	20 (20;20)	27	20 (19;20)
	Control (*N* = 42)							
De Morton Mobility Index (points)	43	58.1 (16.2)	28	64.9 (14.2)	26	69.5 (15.7)	25	65.1 (15.8)
24-h mobility (h/day)								
Lying/sitting	25	21.5 (1.5)	25	18.0 (2.1)	19	20.3 (1.7)	17	19.9 (1.9)
Uptime	25	1.8 (1.1;2.8)	25	3.7 (2.0;4.5)	19	3.4 (2.3;4.2)	17	3.9 (2.7;4.6)
Standing	25	1.7 (1.0;2.1)	25	3.1 (1.5;4.0)	19	2.7 (1.6;3.7)	17	2.9 (2.1;3.8)
Walking	25	0.2 (0.1;0.6)	25	0.5 (0.3;0.6)	19	0.6 (0.5;0.9)	17	0.6 (0.4;0.9)
Steps (*n*/day)	25	754 (187;2352)	25	1961 (1370;2791)	19	2800 (1607;3509)	17	2319 (1300;4092)
Transitions (*n* up-down/day)	25	47 (26;68)	25	46.7 (38;65)	19	51 (36;64)	17	57 (39;69)
Isometric knee extension (Nm/kg)	40	0.6 (0.5;0.8)	27	0.6 (0.4;0.8)	26	0.57 (0.44;0.75)	23	0.6 (0.5;0.7)
30-s Sit-to-stand test (reps)	39	6 (0;9)	28	8.5 (3.5;10)	26	7.5 (5;12)	24	6 (3;11.5)
30-s Sit-to-stand test mod (reps)^a^	10	7.5 (4;9)	4	5.5 (3.5;7)	3	7 (6;9)	4	6 (4;8.5)
Habitual Gait Speed (m/s)	41	0.6 (0.5;0.8)	29	0.7 (0.5;0.8)	26	0.64 (0.54;0.87)	25	0.6 (0.5;0.8)
Hand grip strength (kg)	43	21.1 (8.7)	29	21.8 (8.9)	26	22.4 (8.7)	25	21.8 (8.4)
Barthel Index 20 (points)	43	19 (18;20)	29	20 (19;20)	26	20 (19;20)	25	20 (19;20)

Variables are presented as mean (SD) or median (IQR) depending on the distribution of the variables

*ITT* intention to treat, *reps* repetitions

^a^ Modified version where the use of the armrests is allowed

#### Primary outcome

Both the ITT analysis and the PP analysis showed no significant between-group difference in change in the DEMMI score for any of the three periods assessed (baseline to 4 weeks after discharge; baseline to discharge; 4 weeks to 6 months) (Table 2). In addition, no differences were found when adjusting the analyses for baseline DEMMI score or LOS (results not shown). However, there was a significant change in DEMMI score from baseline to 4 weeks in both groups in both the ITT analysis (mean difference from baseline to 4 weeks: intervention, 8.3 points (95% CI 0.6; 16.0), *p* = 0.04; control, 11.5 points (95% CI 3.5; 19.4), *p* < 0.01) and in the PP analysis (mean difference from baseline to 4 weeks: intervention, 10.6 points (95% CI 0.6; 20.6), *p* = 0.04; control, 11.6 points (95% CI 2.9; 20.3), *p* < 0.01) (results not shown).

#### Secondary outcomes

During hospitalization, the participants spent an average of 21.7 h per day sedentary (sitting or lying), a median of 1.8 h in an upright position (standing or walking) and took a median of 702 steps per day. On admission, their knee extension strength was 0.7 Nm/kg (IQR 0.5; 0.9), they performed six sit-to-stand transitions in 30 s (IQR 0; 9), their habitual gait speed was 0.6 m/s (IQR 0.4; 0.8), handgrip strength was 28.9 kg in men (IQR 23.1;37.0) and 15.6 kg in women (IQR 13.0;20.2), and they had a Barthel score of 20 (IQR 18; 20) (Table 1).

For all secondary outcomes, the ITT analyses showed no significant between-group differences in change scores for any of the three periods (baseline to 4 weeks; hospitalization; post intervention) except for an increase in handgrip strength in the intervention group during hospitalization in both the unadjusted and the adjusted analyses (Table 2). The PP analyses showed that between baseline and 4 weeks post discharge, the daily number of steps taken increased significantly more in the intervention group (difference in change from baseline to 4 weeks, 1033.40 (95% CI 4.13; 2062.66); *p* = 0.049 adjusted for DEMMI score at baseline and length of stay; 1032.77 (95% CI 3.60; 2061.94); *p* = 0.049 adjusted for DEMMI score at baseline, steps at baseline, and length of stay) compared to the control group and increased more in gait speed and handgrip strength during hospitalization (Table 2). Overall, there was a significant increase in STS from baseline to 4 weeks (mean difference from baseline to 4 weeks, overall: ITT analysis, 2.9 (95% CI 1.3; 4.7), *p* = 0.001; PP analysis, 3.0 (95% CI 0.6; 5.3), *p* = 0.01). Also, there was a significant change within both groups in the ITT analysis (mean difference from baseline to 4 weeks: intervention, 3.1 (95% CI 0.5; 5.6), *p* = 0.02; control, 2.7 (95% CI 0.3; 5.2), *p* = 0.03) and in the control group in the PP analysis (mean difference from baseline to 4 weeks: intervention, 3.0 (95% CI −0.8; 6.9), *p* = 0.12; control, 3.0 (95% CI 0.2; 5.9), *p* = 0.04). There were no within-group in gait speed, handgrip strength, knee extension strength, or ADL (results not shown).

### Compliance and satisfaction with the intervention

The majority of the patients started the intervention between 0 and 2 days after admission (78.8%) - range 0–4. Overall, 43% (18/42) of the patients randomized to the intervention group were very compliant with the intervention (80% of sessions performed with two sets of eight RM). Of those who remained in the study at 4 weeks, 60% (18/30) were very compliant and 23% (7/30) were moderately compliant with the intervention (minimum 8 out 12 (67%) sessions performed with two sets of eight RM). All patients consumed the amount of protein stated in the protocol. Between week 1 and week 4 of the intervention, there was a general increase in the level of exercise performed in both the sit-to-stand exercise and the heel-raise exercise. Thus, in the sit-to-stand exercise, 20% more patients trained at levels 6–7 in week 4 compared to week 1, and in the heel-raise exercise, 24% more patients trained at levels 5–7 in week 4 compared to week 1. Also, in both exercises those training with a weighted vest (STAND level 6, heel-raise level 5) increased their load by 1.5 kg (*p* = < 0.01) and 2 kg (*p* < 0.01), respectively. None of the patients reported an increase in pain during the training sessions and no adverse events were reported.

Twenty-five patients from the intervention group were asked about their satisfaction with the intervention. The majority were satisfied or very satisfied (88%) with the intervention, and two thirds (68%) were satisfied or very satisfied with the results of the intervention. The majority (17/25) felt they had benefitted from the strength training sessions and that the amount of training was appropriate (20/25). Also, 16/25 said they would continue training either alone or with others.

There was no difference between the groups in the number of readmissions between discharge and 4 weeks and between discharge and 6 months. Also, there was no difference between the groups in the number of days hospitalized between discharge and 4 weeks and between discharge and 6 months (results not shown).

## Discussion

This randomized controlled trial investigated the efficacy of a simple, supervised, strength training program for the lower extremities, combined with post-training oral protein supplementation, initiated during hospitalization and continued in the home setting for 4 weeks after discharge in older medical patients admitted with acute illness. The main finding was that no effect of the intervention was seen on mobility assessed by the DEMMI score at any of the investigated time points. However, there was a significant increase in the number of steps taken in the intervention group as compared to the control group in our per-protocol analysis. Overall, 43% of the patients were highly compliant with the intervention and there was a general increase in the level of exercise performed in both exercises. None of the intervention group patients reported an increase in pain during the exercises and most of them expressed satisfaction with the intervention.

The intervention was not superior to usual care in improving mobility (DEMMI) from baseline to 4 weeks. However, both groups improved their mobility and the improvement reached beyond a clinically important difference [56] in the control group in the intention-to-treat analysis and in both groups in the per-protocol analysis. Also, there was no effect on mobility during hospitalization. This is in agreement with previous studies in geriatric patients showing no effect on mobility, assessed by the DEMMI score [87], nor on functional outcomes [88] of an in-hospital, progressive, strength training program. Several reasons for the lack of effect on our primary outcome can be suggested.

Firstly, the intervention had a relatively short duration. It is likely that longer training periods are required to benefit older adults. Studies by Hvid et al. [16, 89] have shown that older adults are more susceptible to periods of inactivity and require more time than younger adults to fully recover. Also, Jadczak et al. recommend interventions with a duration of at least 2.5 months [90]. Accordingly, a systematic review by Valenzuela et al. [36] concludes that progressive resistance training in older nursing home residents is efficient in improving strength and functional performance despite advanced age, chronic diseases, sedentary habits, and functional disabilities. However, all interventions investigated by Valenzuela et al. had a duration of at least 2 months [36]. Equivalently, previous studies have identified an effect on both strength and functional abilities of 10 weeks of supervised, progressive, lower extremity resistive exercises using own weight and Therabands in both frail older adults living in a care facility [91] and community-dwelling older adults [92]. Equal to our study, both studies used 3–weekly training sessions, but slightly more extensive programs (4–8 exercises) [91, 92]. A systematic review by Borde et al. looking at resistance training in healthy older adults found that the training period, the intensity, and the total time per repetition under tension are all parameters of significance for the effect on muscle strength with the largest effect sizes seen for the longest periods (50–53 weeks), intensities of 70–79% of 1 RM, and a total time under tension per repetition of 6 s [93]. While the intensity and time under tension recommended by Borde et al. [93] were used in our intervention, we were far from the optimal period presented (50–53 weeks). However, it is questionable whether these recommendations can be followed in newly discharged, older medical patients. Admitted with acute illness. Therefore, we chose a minimal treatment approach focusing on the initial 4 weeks after discharge. Despite a much shorter training period of only 4 weeks, we experienced a substantial drop out rate (28%) between baseline and 4 weeks and less than half of the remaining participants were very compliant with the intervention (43%), indicating that participation would have been even lower in longer interventions.

Secondly, even though the DEMMI has been shown valid and reliable in measuring mobility in both older medical patients and in community-dwelling older adults [53, 54, 56], and has the ability to measure change in mobility after hospital discharge [56], the patients in that validation sample had much poorer mobility than our participants. Only half of the participants had a baseline DEMMI score below 62, reflecting limited mobility. The functions causing most participants difficulties were related to static and dynamic balance, i.e. tandem stand with closed eyes, walking four steps backwards, and jumping [94]. Obtainment of a significant between-group difference in favor of the intervention group would have required that the intervention group participants had learned these three functions, which is highly unlikely based on the provided intervention of sit-to-stand and heel-raise exercises. Thus, for the intervention chosen, the DEMMI score may not have been the best choice of primary outcome. Also, the intervention proposed may not be suitable for all patients, and it is worth considering stratifying patients to different interventions according to their mobility difficulties. Accordingly, a recent umbrella review [90] investigating the effect of exercise interventions in pre-frail, community-dwelling, older adults found inconclusive results in the effect on mobility of both multi-component exercises and resistance exercises and suggested that only personalized exercises are effective in improving mobility.

Overall, we found no or very few between-group differences for the secondary trial outcomes. We found a difference in hand grip strength and gait speed during hospitalization in favor of the intervention group and a difference in the change in daily number of steps taken between baseline and 4 weeks in our per-protocol analysis. Previous studies have also reported improvement in functional performance measures during hospitalization [95, 96]. However, in accordance with these studies [95, 96] the patients in the present study had poor performance at discharge - their knee extension strength was at the threshold level for independent ability to perform activities of daily living [97], putting them at increased risk of future mobility limitations [98]. Also, their hand grip strength and walking speed were at levels indicating mobility limitations [69]. These low levels of functional performance at discharge are worthy of concern, since functioning has been linked with future risk of falls, functional decline [99], mobility and ADL disability [68, 100, 101], hospital readmissions [28], and death [67, 99, 102]. Older adults see mobility related to everyday functioning as vital to their health and as an indicator of wellbeing and independence enabling them to participate in life as they know it and therefore affecting more that the physical aspects of their life [103]. This underlines the importance of trying to counteract further mobility decline in connection with hospitalization, to help older adults maintain their independence. Like our study, Tibaek et al. [88] found no effect on mobility or of strength training in addition to standard physiotherapy. Also, Oestergaard et al. [87] found no effect of an in-hospital, chair-based, exercise program on mobility and muscle strength.

During hospitalization the participants took a median of 700 steps daily. This small number of steps during hospitalization has been linked to hospital-associated functional decline [104]. Acknowledging that this was a secondary finding, we observed a significant between-group difference in change in the number of steps taken between baseline and 4 weeks in favor of the intervention group in our per-protocol analysis. At 4 weeks the control group took a median of 2800 steps compared to 4100 in the intervention group (Table 3). This difference is promising, since Floegel et al. [105] found that each 1000 additional steps in the post-discharge period in older women with heart failure was associated with better physical performance. Also, Breen et al. [22] found that reducing the daily number of steps in healthy older adults by 1400/day over a fortnight led to a 4% reduction in leg lean mass, modest increases in inflammation markers, and altered insulin sensitivity. Although the present study did not show an effect on either mobility or functional performance measures, the effect on the number of steps taken is promising. Merely focusing on enhancing the number of steps taken during hospitalization and post discharge could be a goal for future studies, thus lowering the requirements for acutely ill older adults who might find themselves unable to exercise [29]. In addition, an increase in the number of steps taken during hospitalization is associated with shorter length of stay [106], while a decline is associated with greater risk of death within 2 years after discharge [107]. Accordingly, an association has been shown between steps per day post discharge and 30-day readmission rate [108]. In addition, Brown and co-authors [109] found that factors like weakness, need for assistance, lack of interest from staff, and structural barriers are reasons for inactivity in older medical patients (≥ 75 years). Thus, based on the findings from the present trial, we have changed our focus from exercise to walking, to increase intervention compliance. In an ongoing study, we investigate the effect of an intervention including a physical component (i.e. promoting walking) and a component focusing on overcoming structural barriers [110].

### Strengths and limitations

Study strengths included that our intervention was initiated at the hospital and continued at home a few days after discharge, as well as choosing a minimally time-consuming treatment approach taking into account its implementation in a busy care-setting. Acutely hospitalized older adults may prefer exercise to be initiated in the hospital or shortly after discharge. Also, Franco and co-authors found that exercise at home, an improvement in the ability to undertake daily tasks, and not having to use transportation were the three most important attributes for engaging in physical activity among community-dwelling older adults with a history of falls or self-reported mobility disability [111]. Thus, the fact that our invention took place in the participants’ own homes shortly after discharge may have enhanced compliance. However, we do not have data to support this hypothesis.

Another strength of our study is that we have tried to overcome the previously reported lack of knowledge on the optimal nature and dose of exercise in older adults [32, 38, 42]. According to a recent review [112], low intensities are often the first choice among physiotherapists, as this is perceived to be safer. Low intensities, though, may be inadequate to achieve optimal effects on functional performance [41], which is why we wanted to investigate whether higher intensities could be performed by older medical patients without inducing adverse events. Since we found few studies investigating the effect of a cross-continuum program initiated during hospitalization and continued after discharge [29, 43], and due to problems with compliance in these studies, we chose a program with full supervision from trained staff. A 4-week period was chosen since it has previously been reported that recovering function within the first month after discharge is of importance for long-term outcomes [27]. Also, a study in older clients in home care found that structured exercise programs are not the preferred activity of these older adults [113], which is why a 4-week program may be more acceptable than a program of longer duration. Additionally, a previous study in older hospitalized adults showed positive effects of exercise therapy performed during the first 4 weeks after discharge [43], leading us to believe that 4 weeks might be sufficient in inducing an effect. Protein was chosen as an integrated part of the strength training intervention. Both resistance training and amino acids can stimulate an anabolic response [114] and the two in combination have been shown to enhance the muscular response to exercise in healthy older adults [45–47]. The provided protein supplementation was intended to boost anabolism. However, it is unclear whether it has merely reduced an existing protein deficit since less than half of our participants could be considered to have a normal nutritional state on admission. Nevertheless, since older adults need a greater amount of daily protein than young adults to maintain muscle mass, and older adults with acute or chronic diseases or marked malnutrition, need even more [115], our intention was to fill out a potential protein gap.

A limitation in our study was that a substantial number of participants dropped out between baseline and 4 weeks (27%) - half of these (*N* = 12) dropped out between the admission and the discharge assessment. However, between baseline and 4 weeks the number of drop outs was smaller in the intervention group than in the control group, indicating that the intervention itself was not the reason for dropping out. This is in line with previous studies reporting equivalent drop-out rates for in-hospital [88] and post-discharge training interventions [116]. Furthermore, at baseline there was no significant difference in the DEMMI score between those who remained in the study and those who dropped out. In the present study, participants in both groups choosing to refrain from further participation said they lacked time and felt that the main entrance of their house had turned into a revolving door of health professionals. Also, older medical patients that may benefit from physical rehabilitation during and after hospitalization may have barriers preventing exercise participation. In a study by Brown et al. [29] reporting difficulties in recruiting acutely admitted older medical patients, those declining to participate expressed that they did not feel like exercising or did not believe they could. The reasons for non-participation in the present study were e.g. lack of time, believing oneself to be sufficiently active, or disbelief in the effect of exercise. A large number of patients were excluded from our study or declined to participate, leaving us with a very selective group of older medical patients. However, similar or lower consent rates have been reported in previous studies in older medical patients [29, 43, 117]. This underlines the difficulties in recruiting patients in the acute setting and limits the generalizability of the results. In the intervention group, only 43% were very compliant with the intervention, which may explain the lack of effect seen in this study. This level of compliance, though, is in line with a study in acutely admitted geriatric patients [87] and stresses the challenges in maintaining acutely admitted older adults in training interventions. Also, we experienced a large amount of missing data in our study, due among other reasons to the high number of drop outs. In addition, several ActivPals were lost during the intervention period, which may have affected the results. Furthermore, a limitation of our study was that we did not measure the rate of perceived exertion as an indicator of performance intensity. However, in a feasibility study preceding the present study, we found that the subjectively perceived effort, assessed by the BORG score, when performing the exercises used in the present study, corresponded well with our aim of 8–12 repetitions being equivalent with 60–70% exertion [51]. Thus, perceived exertion was not assessed in the present study.

## Conclusions

A simple, low technology, supervised strength training program for the lower extremities, combined with post-training oral protein supplementation initiated during hospitalization and continued in the home setting for 4 weeks after discharge, was not superior to usual care in the effect on change in mobility 4 weeks after discharge in older medical patients admitted with acute illness. For the secondary outcome, the daily number of steps, good compliance with the intervention resulted in a greater daily number of steps.

## Supplementary information

**Additional file 1.** Number of imputations at different time points for the primary and secondary outcomes. The table shows the number of imputations used at each assessment point for the primary and the secondary outcomes for both the intention-to-treat analysis and the per-protocol analysis.

## Data Availability

The datasets generated and/or analyzed during the current study are not publicly available due to regulations set out by the Danish Data Protection Agency regarding data anonymization but are available from the corresponding author on reasonable request.

## Abbreviations

ADL: Activities of daily living
BI: The Barthel Index 20
CI: Confidence interval
CONSORT: Consolidated Standards of Reporting Trials
COPD: Chronic obstructive pulmonary disease
DEMMI: The De Morton Mobility Index
Heel-raise: Heel raise exercise
HG: Habitual gait speed
HGS: Hand-grip strength
ICD-10: The International Classification of Diseases, 10th Edition
IQR: Interquartile range
ITT: Intention to treat
LOS: Length of stay
NMS: New Mobility Score
PP: Per protocol
RM: Repetition maximum
SD: Standard deviation
STAND: Sit-to-stand exercise
STS: The 30-s sit-to-stand test
VRS: The Verbal Ranking Scale
