# Supplementary material for: A randomized controlled trial of the effect of supervised progressive cross-continuum strength training and protein supplementation in older medical patients: the STAND-Cph trial
Source: Trials. 2019 Nov 28;20:655. doi: 10.1186/s13063-019-3720-x (PMC6883554; doi:10.1186/s13063-019-3720-x)
Supplement: Supplementary file 1 — Additional file 1. Number of imputations at different time points for the primary and secondary outcomes. The table shows the number of imputations used at each assessment point for the primary and the secondary outcomes for both the intention-to-treat analysis and the per-protocol analysis. [file 13063_2019_3720_MOESM1_ESM.docx]

Additional file 1. Number of patients with imputed values at different time points for the primary and secondary outcomes

| **Outcomes in intention to treat analysis** | **Assessment** | | | |
| --- | --- | --- | --- | --- |
|  | **Baseline** | **Discharge** | **4 weeks** | **6 months** |
| DEMMI | No patients (0 %) | 20 patients (23.5 %)  *5 intervention*  *15 control* | 29 patients (34.1 %)  *12 intervention*  *17 control* | 33 patients (38.8%)  *15 intervention*  *18 control* |
| Knee extension strength | 3 patients at baseline (3.5 %)  *3 control* | 22 patients (25.9 %)  *6 intervention*  *16 control* | 30 patients (35.8 %)  13 intervention  17 control | 36 patients (42.4 %)  *16 intervention*  *20 control* |
| Sit to stand | 6 patients (7.1 %)  *2 intervention*  *4 control* | 20 patients (23.5 %)  *5 intervention*  *15 control* | 29 patients (34.1 %)  *12 intervention*  *17 control* | 34 patients (40.0 %)  *15 intervention*  *19 control* |
| Gait speed | 3 patients (3.5 %)  *1 intervention*  *2 control* | 19 patients (22.4 %)  *5 intervention*  *14 control* | 29 patients (34.1 %)  *12 intervention*  *17 control* | 33 patients (38.8%)  *15 intervention*  *18 control* |
| Hand grip | No patients (0 %) | 18 patients (21.2 %)  *4 intervention*  *14 control* | 29 patients (34.1 %)  *12 intervention*  *17 control* | 33 patients (38.8%)  *15 intervention*  *18 control* |
| Barthel | No patients (0 %) | 19 patients (22.4 %)  *5 intervention*  *14 control* | 29 patients (34.1 %)  *12 intervention*  *17 control* | 33 patients (38.8%)  *15 intervention*  *18 control* |
| 24-hour activity | 24 patients (28.2 %)  *6 intervention*  *16 control* | 29 patients (34.1 %)  *11 intervention*  *18 control*  11 patients with partial imputations (12.9 %)  *2 from day 2 (1 int., 1con.)*  *1 from day 3 (1 int.)*  *2 from day 4 (1 int., 1 con.)*  *2 from day 5 (2 con.)*  *4 from day 6 (2 int., 2 con.)* | 41 patients (48.2 %)  *17 intervention*  *24 control*    11 patients with partial imputations (12.9 %)  *2 from day 3 (2 int.)*  *1 from day 4 (1 int.)*  *2 from day 5 (1 int., 1 con.)*  *6 from day 6 (4 int., 2 con.)* | 46 patients (54.1 %)  *20 intervention*  *26 control*  10 patients with partial imputations (11.8 %)  *1 at day 2 (1 int.)*  *2 at day 3 (1 int., 1con.)*  *1 at day 4 (1 con.)*  *3 at day 5 (1 int., 2 con.)*  *3 at day 6 (2 int., 1 con.)* |
| **Outcomes in per protocol analysis** | **Baseline** | **Discharge** | **4 weeks** | **6 months** |
| DEMMI | No patients (0 %) | 3 patients (3.5 %)  *3 control* | No patients (0 %) | 3 patients (3.5 %)  *2 intervention*  *1 control* |
| Knee extension strength | 1 patient (1.2 %)  *1 control* | 4 patients (4.7 %)  *1 intervention*  *3 control* | No patients (0 %) | 5 patients (5.9 %)  *2 intervention*  *3 control* |
| Sit to stand | 2 patients (2.4 %)  *1 intervention*  *1 control* | 3 patients (3.5 %)  *3 control* | No patients (0 %) | 4 patients (4.7 %)  *2 intervention*  *2 control* |
| Gait speed | 1 patient (1.2 %)  *1 control* | 2 patients (2.4 %)  *2 control* | No patients (0 %) | 3 patients (3.5 %)  *2 intervention*  *1 control* |
| Hand grip | No patients (0 %) | 2 patients (2.4 %)  *2 control* | No patients (0 %) | 3 patients (3.5 %)  *2 intervention*  *1 control* |
| Barthel | No patients (0 %) | 2 patients (2.4 %)  *2 control* | No patients (0 %) | 3 patients (3.5 %)  *2 intervention*  *1 control* |
| 24-hour activity | 8 patients (9.4 %)  *1 intervention*  *7 control* | 6 patients (7.1 %)  *2 intervention*  *4 control*  7 patients with partial imputations (8.4 %)  *1 at day 2 (con.)*  *1 at day 4 (int.)*  *1 at day 5 (con.)*  *4 at day 6 (2 int., 2 con.)* | 10 patients (11.8 %)  *3 intervention*  *7 control*  7 patients with partial imputations (8.4 %)  *1 at day 3 (int.)*  *1 at day 5 (con.)*  *5 at day 6 (3 int., 2 con.)* | 14 patients (16.5 %)  *5 intervention*  *9 control*  4 patients with partial imputations (4.7 %)  *1 at day 2 (int.)*  *2 at day 3 (1 int.,1 con.)*  *1 at day 4 (1 con.)* |

*The table shows the number of imputations made at the 4 assessment points.The distribution of imputations on intervention group patients and control group patients is shown in italic. Control/con.: Control group; Intervention/int.: Intervention group;Partial imputation: Partial imputations were carried out for patients with missing data on some of the 7 assessed days of assessment (e.g. if the ActivPal monitor has fallen off on day 4, imputations would be made for days 4 to 7).*
